# Isolation and Characterization of Engineered Nucleoside Deoxyribosyltransferase with Enhanced Activity Toward 2’-Fluoro-2’-Deoxynucleoside

**DOI:** 10.4014/jmb.2204.04041

**Published:** 2022-06-15

**Authors:** Yeon-Jin Yoo, Kang-Hyun Choi, Byoung-Kyun Kim, Si-Sun Choi, Eung-Soo Kim

**Affiliations:** 1 Department of Biological Sciences and Bioengineering, Inha University, Incheon 22212, Republic of Korea; 2 Division of Bioprocess Discovery, ST Pharm, Gyeonggi-do 15610, Republic of Korea

**Keywords:** Nucleoside deoxyribosyltransferase, 2’-fluoro-2’-deoxynucleoside, error-prone PCR, substrate specificity, enzyme kinetics

## Abstract

Nucleoside deoxyribosyltransferase (NDT) is an enzyme that replaces the purine or pyrimidine base of 2′-deoxyribonucleoside. This enzyme is generally used in the nucleotide salvage pathway in vivo and synthesizes many nucleoside analogs in vitro for various biotechnological purposes. Since NDT is known to exhibit relatively low reactivity toward nucleoside analogs such as 2’-fluoro-2’-deoxynucleoside, it is necessary to develop an enhanced NDT mutant enzyme suitable for nucleoside analogs. In this study, molecular evolution strategy via error-prone PCR was performed with *ndt* gene derived from *Lactobacillus leichmannii* as a template to obtain an engineered NDT with higher substrate specificity to 2FDU (2′-fluoro-2′-deoxyuridine). A mutant library of 214 *ndt* genes with different sequences was obtained and performed for the conversion of 2FDU to 2FDA (2′-fluoro-2′-deoxyadenosine). The *E. coli* containing a mutant NDT, named NDT^L59Q^, showed 1.7-fold (at 40°C) and 4.4-fold (at 50°C) higher 2FDU-to-2FDA conversions compared to the NDT^WT^, respectively. Subsequently, both NDT^WT^ and NDT^L59Q^ enzymes were over-expressed and purified using a His-tag system in *E. coli*. Characterization and enzyme kinetics revealed that the NDT^L59Q^ mutant enzyme containing a single point mutation of leucine to glutamine at the 59^th^ position exhibited superior thermal stability with enhanced substrate specificity to 2FDU.

## Introduction

Oligonucleotide is a potential therapeutic agent with anticancer or antiviral effects. Types of oligonucleotides include antisense oligonucleotides (ASOs), aptamers, small-interfering RNAs (siRNAs), micro-RNAs, antagomir, decoys that bind to transcription factors, and CpG oligonucleotides that activate the immune system [1]. Drugs with various mechanisms of action have been developed using these oligonucleotides. These oligonucleotide treatments are injected into cells in the form of nucleosides because of the difficulty in passing through the cell membrane while charged [2]. Thermal stability and nuclease resistance are important for ASOs to reach mRNA in target cells and exert activity. To alleviate this problem, studies have been conducted to increase the thermal stability and resistance to nuclease by attaching or substituting a specific compound to the sugar part of nucleoside in a site-specific manner [2-6]. In particular, 2′-fluoro-2′-deoxynucleoside is attracting attention as a raw material for the treatment of oligonucleotide because stability against nuclease is high and the melting temperature increases by 1.8°C during the formation of RNA duplexes [7, 8]. Moreover, 2′-fluoro-2′-deoxynucleoside may be synthesized by an enzyme called N-deoxyribosyl transferase (NDT) derived from *Lactobacillus leichmannii* or using purine phosphorylase and pyrimidine phosphorylase. NDT (E.C. 2.4.2.6) is a key enzyme used in the nucleoside salvage pathway [9]. It catalyzes the cut of the glycosidic bond of deoxyribonucleosides and the transfer of the deoxyribosyl moiety to an acceptor purine or pyrimidine base [10].

**Fig. 1:**
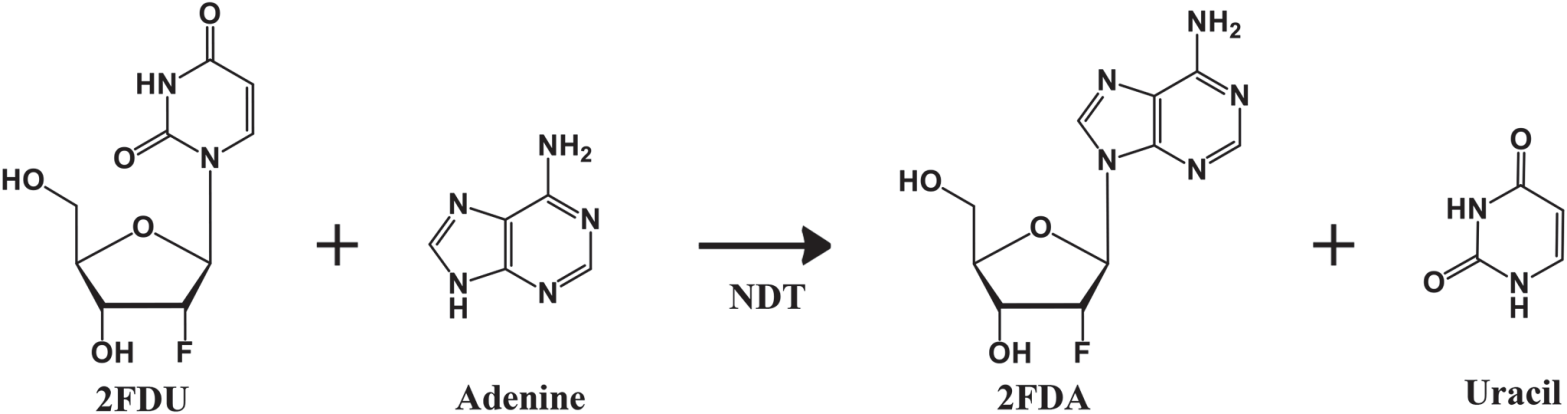
Scheme of NDT conversion reaction with 2’-Fluoro-2’-deoxyuridine (2FDU) to 2’-Fluoro-2’- deoxyadenosine (2FDA).

Currently, there are two types of enzymes: DRTase (Deoxyribosyltransferase) I and DRTase II. DRTase I catalyzes the transfer reaction of deoxyribose between two purines. NDT from *L. leichmannii* is DRTase II and catalyzes the transfer reaction of deoxyribose between purines or pyrimidine [11-13]. DRTase II class NDT also hydrolyzes nucleosides into bases and deoxyribose in the absence of receptor bases [14]. In addition, it has low specificity for receptor bases and stereospecificity that produces only the β-anomer during sugar transition reactions. Therefore, *ndt* derived from *L. leichmannii*, a DRTase II class enzyme, has been used for biosynthesis of many antiviral or anticancer nucleoside analogs [15]. Since previously known NDT exhibits relatively low conversion yield and substrate specificity to fluorine-attached substrate, improvement of NDT substrate specificity to 2′-fluoro-2′-deoxynucleoside as well as its stable over-expression has become a critical issue for biotechnological perspective (Fig. 1).

Here, we employed a molecular evolution strategy to generate an NDT mutant library via error-prone PCR, a technique to induce errors and mismatches to cause random mutation within the target gene [16, 17]. Among the mutants tested, one engineered NDT exhibited much higher activity toward 2FDA than the wild type. Subsequent studies on characterization and enzyme kinetics also revealed that this engineered enzyme exhibited superior thermal stability toward 2′-fluoro-2′-deoxynucleoside.

## Materials and Methods

### Error-Prone PCR for NDT Mutant Library

Nucleoside deoxyribosyltransferase gene (*ndt*) was derived from *Lactobacillus leichmannii* (KCCM35470). Random mutagenesis was performed using an error-prone PCR technique (TaKaRa, Japan) to generate a mutation in the *ndt* gene. The error frequency was set by adjusting the MnSO_4_ and dGTP concentrations in the PCR tube. All constructed plasmids and primers used in this study are described in Fig. S1 and Table S1, respectively. Error-prone PCR was performed by inserting NdeI at the forward primer position on the initiation codon site and XbaI at the reverse primer position on the termination codon site of the gene. The PCR compositions were comprised of 34 μl PCR-grade water, 5 μl 10x TITANIUM *taq* buffer, 640 μM MnSO_4_, 120 μM dGTP, 1 μl 50x diversify dNTP mix, 10 μM of each primer mix (NDTf, NDTr), 1 ng template DNA (pFRPT_*ndt*), and 1 μl TITANIUM *taq* polymerase. The final reaction volume was 50 μl. The PCR cycle was 94°C for 30 s, 55°C for 30 s, 68°C for 30 s, and 68°C for 30 s for 25 cycles. After the PCR reaction, the amplified 474-bp PCR product was extracted by agarose gel electrophoresis. That product was digested with the restriction enzymes XbaI and NdeI. Then, it was cloned into the expression vector, pFRPT_*punp* (purine nucleoside phosphorylase). pFRPT_*punp* (ST Pharm Co. Ltd., Korea) was digested with the restriction enzymes, XbaI and NdeI, to remove the *punp* gene. The digested vector was treated with calf intestinal alkaline phosphatase (CIAP) to prevent self-ligation. The purified PCR product and expression vector digested with XbaI and NdeI were both ligated using T4 ligase. The ligation mixture was transformed to *Escherichia coli* DH5α using a gene pulser. Then, 1 ml of SOC medium was added and cultured for the recovery step at 37°C for 1 h while shaking. The SOC medium included 2% tryptone, 0.5% yeast extract, 10 mM sodium chloride, 2.5 mM potassium chloride, 10 mM magnesium, 10 mM magnesium sulfate, and 20 mM glucose. The culture broth was plated on an LB agar plate containing kanamycin and then further cultured at 37°C for 20 h. The inserted *ndt* gene was verified by colony PCR selecting the transformed colony after ligation to another pFRPT vector using SapphireAmp Fast PCR Master Mix (TaKaRa). The composition and reaction information of the substrate included 5 μl SapphireAmp Fast PCR Master Mix, 10 μM of each primer mix (pFRPTf, pFRPTr), 3 μl dH_2_O, and each of the picked colonies. Ten colonies were randomly selected for sequencing.

### NDT Mutant Library Screening

NDT^WT^ as a control and NDT mutant library were inoculated in 2 ml of LB broth containing kanamycin to culture overnight at 37°C while shaking. Then, 200 μl of the overnight culture solution was inoculated in 20 ml of LB broth containing kanamycin. After 4 h, 20 μl of 1M IPTG (Isopropyl β-D-1-thiogalactopyranoside) was added to perform overexpression followed by culturing for an additional 20 h at 37°C while shaking. The culture medium was centrifuged at 2,600 *×*g and resuspended in 0.5 ml of distilled water. The optical density was measured to determine the input amount of the enzyme-substrate reaction at OD_600_. The measurement was carried out using a UV-Vis spectrophotometer. A 2FDA conversion reaction was conducted for NDT mutant library screening. A reaction sample was prepared with a composition of 1 mM 2FDU, 1 mM adenine, 1 mM potassium phosphate buffer (pH 5.8), and 0.3 OD_600nm_ NDT mutant wet cell. The final reaction volume was 1 ml. The reaction mixture was incubated at 40°C for 20 h while shaking. The reaction was completed, diluted 10 times with 0.1 N NaOH, and then diluted another 10 times with distilled water. The diluted solution was filtered through a 0.45-μm membrane to prepare HPLC samples. The samples were stored at 4°C before the HPLC assay.

### NDT^L59Q^ Activity Confirmation

A 2FDU-to-2FDA conversion assay was prepared with 1 mM 2FDU, 1 mM adenine, 1 mM phosphate buffer (pH 5.8), and 0.3 OD_600_ NDT^WT^ or NDT^L59Q^ wet cells. The reaction was performed at 40 or 50°C for 20 h with shaking. All measurements were performed at least in triplicate. Assay by HPLC was conducted using an ODS-3 column 4.6 × 150 mm, 5 μm; (GL Science, Japan) with detection at 254 nm. The mobile phase was comprised of 0.1% AcOH (acetic acid) and MeOH (methanol) at a 9:1 ratio. Chromatography was performed using an isocratic solution. The elution condition had a flow rate of 1 ml/min and a 20 min run time at 30°C. The major retention times of the standard were RT 2.4 min for adenine, RT 6.16 min for 2'-fluoro-2'-deoxyuridine (2FDU), and RT 12.66 min for 2'-fluoro-2'-deoxyadenosine (2FDA) (Fig. S2).

### NDT Enzyme Expression and Purification in *E. coli*

In the previous *ndt* mutant screening step, NDT^L59Q^, which showed the highest conversion rate, was purified to observe its activity. NDT^WT^ and NDT^L59Q^ were purified through the pET vector system. Each gene sequence was subcloned into the pUC19 vector. Then, each gene was cloned with pET28a (+) and pET21b (-) vectors using an In-fusion HD Cloning Kit (TaKaRa). Each pET vector containing the *ndt* gene was transformed into *E. coli* DH5-a. Subsequently, transformation was performed on *E. coli* BL21 (de3) for protein expression and purification. Pre-culture was conducted in 5 ml of LB broth containing kanamycin or ampicillin at 37°C while shaking. The main culture was conducted in 100 ml of LB broth containing kanamycin or ampicillin. The cells were grown until the OD_600_ reached 0.6 to 0.8 and then they were cooled for 30 min. After cooling, protein overexpression was induced by adding 160 μl of 0.5 M IPTG (Isopropyl β-D-1-thiogalactopyranoside). The final IPTG concentration was 0.8 mM. Overnight induction was then performed at 14°C while shaking. The cells were harvested by centrifugation at 2,600 *×*g. The pellet was resuspended in NPI-10 buffer, and the cells were disrupted by sonication. Sonication was performed at 70% amplitude and pulsed on 10 s, off 15 s for 15 cycles. The disrupted cell debris was centrifuged at 2,600 *×*g for 20 min at 4°C. The supernatant was collected and mixed with Ni-NTA agarose (Qiagen, Germany) for 2 h to purify the protein by affinity chromatography. The buffers used included an equilibrium buffer (NPI-10), a washing buffer (NPI-20), and an elution buffer (NPI-300). The NPI-X buffer contained 50 mM NaH_2_PO_4_•2H_2_O, 300 mM NaCl, and X mM imidazole, and the pH was adjusted to 8.0 using NaOH and HCl. After the purification process, the purified proteins were concentrated using an Amicon Ultra-4 Centrifugal Filter Unit (Merck, Germany) and stored in a desalting buffer (pH 7.2). The desalting buffer contained 50 mM NaH_2_PO_4_•2H_2_O, 1 mM EDTA, and 0.2 mM DTT, and had a final glycerol concentration of 1%. The protein expression was confirmed by SDS-PAGE. The protein concentration was measured using a Bradford assay.

### Characterization and Kinetics of NDT Mutant Enzyme

The optimal reaction time was determined by measuring the production of 2FDA. Two purified enzymes, N-terminal 6x His-tagged NDT^WT^ and NDT^L59Q^, were added into the reaction. The composition of the reaction solution (100 μl) was 1 mM 2FDU, 1 mM adenine, 1 mM potassium phosphate buffer (pH 5.8), and 0.1 mg/ml of each enzyme. The optimal pH of each enzyme activity was studied using 10 mM 2FDU, 10 mM adenine, 10 mM sodium citrate buffer (pH 5), 10 mM potassium phosphate buffer (pH 6.0 to 8.0), and 0.1 mg/ml of each NDT enzyme. The pH of the sodium citrate buffer was adjusted by adding HCl and NaOH. The potassium phosphate buffer was obtained by adjusting the potassium phosphate dibasic-to-monobasic ratio. An experiment was conducted to find the optimal temperature for the substrates to react into 2FDU using the obtained NDT^L59Q^ and NDT^WT^. The reaction condition of this experiment was 1 mM 2FDU, 1 mM adenine, 1 mM potassium phosphate buffer (pH 5.8), and 0.1 mg/ml of each NDT enzyme. The reaction was performed for 20 h at 30 to 60°C. In addition, a thermal stability test was performed. The enzymes were preheated for five minutes at each temperature point from 40 to 80°C. The substrate reactions were performed with 10 mM 2FDU, 10 mM adenine, 1 mM potassium phosphate buffer (pH 5.8), and 0.1 mg/ml of each NDT enzyme. The reacted samples were diluted 10-fold in 0.1 N NaOH and then stopped by storing at 4°C before HPLC. The substrates for NDT enzyme kinetics were prepared using a master mixture containing 10 mM 2FDU, 10 mM adenine, and 10 mM phosphate buffer, diluted to 1, 2, 4, 6, 8, and 10 mM. Then, 0.1 mg/ml of each enzyme was put into the reaction. The final reaction volume was 100 μl and was reacted at pH 6, and 40°C for 20 h. The reacted sample was stopped by diluting the sample 10-fold in 0.1 N NaOH and then it was stored at 4°C for HPLC. Six measurements were taken at each substrate concentration (1 mM to 10 mM). A graph was created with four experimental data points, excluding the two highest and lowest values. The obtained data (displayed as an error bar) were reversed and shown on the graph, and a Lineweaver–Burk plot was drawn to obtain the *K*_m_ and *V*_max_ values. Their means and standard deviations were obtained, and their values were listed in Table 2.

## Results and Discussion

### NDT Mutant Library Screening and Isolation

**Fig. 2:**
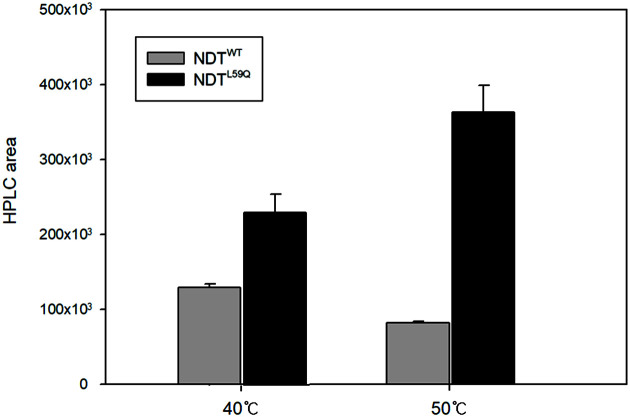
2FDU-to-2FDA conversion by *E. coli* whole cell containing NDT^WT^ (gray) and NDT^L59Q^ (black). Reaction was performed at 40°C and 50°C for 20 h. Substrate concentrations of 1 mM were prepared.

Ten random colonies were screened for sequencing. They have one-to-three-point mutations per clone. The reaction was carried out through whole-cell conversion with 214 mutant libraries obtained. At the same time, NDT^WT^ was used as a control. The activity of NDT, which is the 2FDA generation reaction conversion rate, was evaluated from the ratio of the amount of the product to the amount of the substrate. It is expressed as follows: 2FDA generation reaction conversion rate = [100 * 2FDA (mole) / {(2FDA (mole)+2FDU (mole)}]. Most of the colonies exhibited less than 1.0 titer compared to control. Some colonies showed 1.31 times the conversion rate compared to the control even though no mutation occurred (Table 1). In the 2FDU-to-2FDA conversion reaction, the colony with the highest conversion rate was C71, where 176^th^ thymine was substituted with adenine, and 59^th^ leucine was substituted with glutamine (named as NDT^L59Q^). NDT^L59Q^ mutant showed a 2FDA production yield about 1.7 times at 40°C and 4.4 times at 50°C higher than the control NDT^WT^ (Fig. 2). Multiple sequence alignment was conducted with strains with other NDTs to determine the correlation of the site where the mutation occurred. A mutation occurred in the preserved site in the amino acid at the 59^th^ location in the *ndt* gene, substituted from leucine with a hydrophobic side chain with glutamine with a polar uncharged side chain is believed to play an important role in the exceptionally high specificity of NDT^L59Q^ for 2′-fluoro-2′-deoxynucleoside (Fig. S3).

### NDT Gene Expression and Enzyme Characterization

**Fig. 3:**
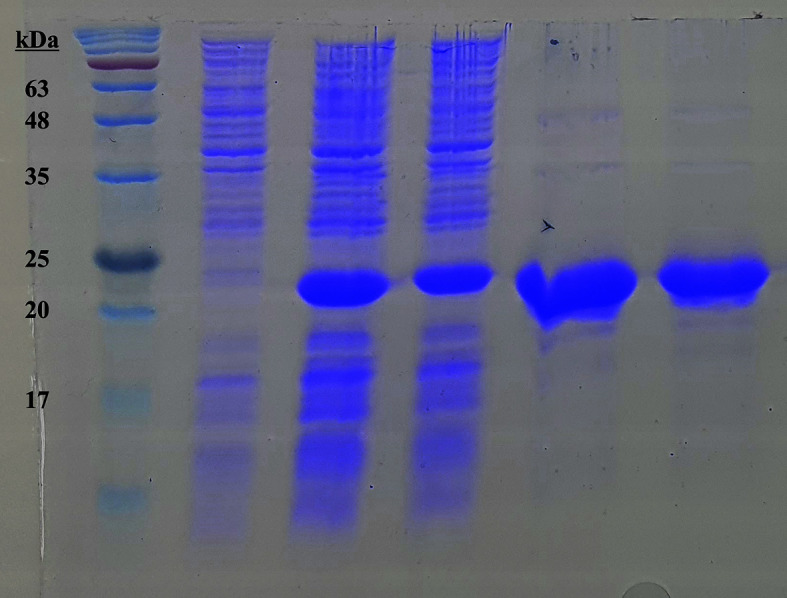
SDS-PAGE of purified N-terminal 6x His-tagged NDT^WT^ and NDT^L59Q^ enzymes. Lane 1: Dyne bio ladder molecular weight marker; Lane 2: *E. coli* BL21 (de3) pET28a total; Lane 3: *E. coli* BL21 (de3) pET28a_ndt^WT^ total; Lane 4: *E. coli* BL21 (de3) pET28a_ndt^L59Q^ total; Lane 5: purified NDT^WT^ protein; Lane 6: purified NDT^L59Q^ protein. SDS-PAGE confirmed that the protein spread over 15% polyacrylamide gel appeared near 20.2 kDa where the band was not visible in *E. coli* BL21 (de3), which contains a controlled pET28a vector.

**Fig. 4:**
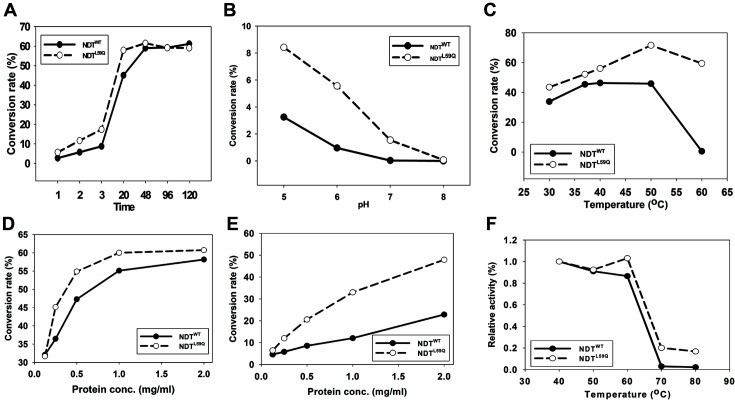
Various effects on NDT activities. (**A**) Time, (**B**) pH, (**C**) Temperature, (**D**) Protein concentration in 1 mM substrate, (**E**) Protein concentration in 10 mM substrate, (**F**) Heat stability.

The gene coding ndt^WT^ or ndt^L59Q^ mutant was cloned into pET28a (+) and pET21b (-), respectively, to overexpress NDT. SDS-PAGE confirmed that the protein spread over 15% polyacrylamide gel appeared near 20.2 kDa where the band was not visible in *E. coli* BL21 (de3), which contains a controlled pET28a (+) vector (Fig. 3, Lane 2). Although the *ndt* cloned to pET21b (-) containing the His-tagging at the C-terminal was not expressed, ndt^WT^ or ndt^L59Q^ cloned to pET28a (+) with the His-tagging at the N-terminal was well expressed (Fig. 3, Lanes 3, 4) and purified as a single band (Fig. 3, Lanes 5, 6). To observe the enzyme properties in more detail, purified NDT was characterized. The conversion rate increased linearly up to the first three hours of the reaction. After 20 h, the conversion rate was relatively constant until it reached 120 h (Fig. 4A). The stability of the two enzymes for pH was evaluated by preparing a reaction buffer containing a substrate in the range of pH 5.0 to 8.0. In the case of NDT^WT^, there is only a low conversion rate when the pH is increased above 7.0, whereas there is a noticeable conversion rate in the case of NDT^L59Q^. A significant conversion rate cannot be obtained at a pH of 7.0 or higher (Fig. 4B). The optimal temperature of the enzyme was obtained by performing a reaction at 30 to 60°C. From 37 to 50°C, both enzymes showed relatively high conversion rates. On the other hand, at 60°C, there was a low conversion rate of NDT^WT^, while with NDT^L59Q^, the conversion rate was higher than 60% (Fig. 4C). The conversion rate according to the enzyme amount was shown at two substrate concentrations of 1 mM and 10 mM. At a substrate concentration of 1 mM, the conversion rate increased to 2.0 mg/ml of enzyme in NDT^WT^. In the case of NDT^L59Q^, the conversion rate no longer increased when the enzyme concentration was 1.5 mg/ml or more. At a substrate concentration of 10 mM, the conversion rate increased linearly with increasing enzyme concentration. This indicates that in the case of NDT^L59Q^, the maximum conversion rate can be reached with a small amount of enzyme. The higher conversion rate of NDT^L59Q^ is shown in Figs. 4D and 4E imply that the higher activity of the NDT^L59Q^ mutant might be derived from the better tolerance toward higher substrate range of 2'-fluoronucleoside, of which the mechanism needs to be further pursued. A heat stability activity experiment on the enzyme was conducted. The y-axis value was set to the relative activity to compare the thermal stability. NDT^L59Q^ had a relative activity of 20% and 16.8% at 70°C and 80°C, respectively. On the other hand, NDT^WT^ had a relative activity of 3%and 2% at the same temperature, respectively (Fig. 4F).

### NDT Enzyme Kinetics

**Fig. 5:**
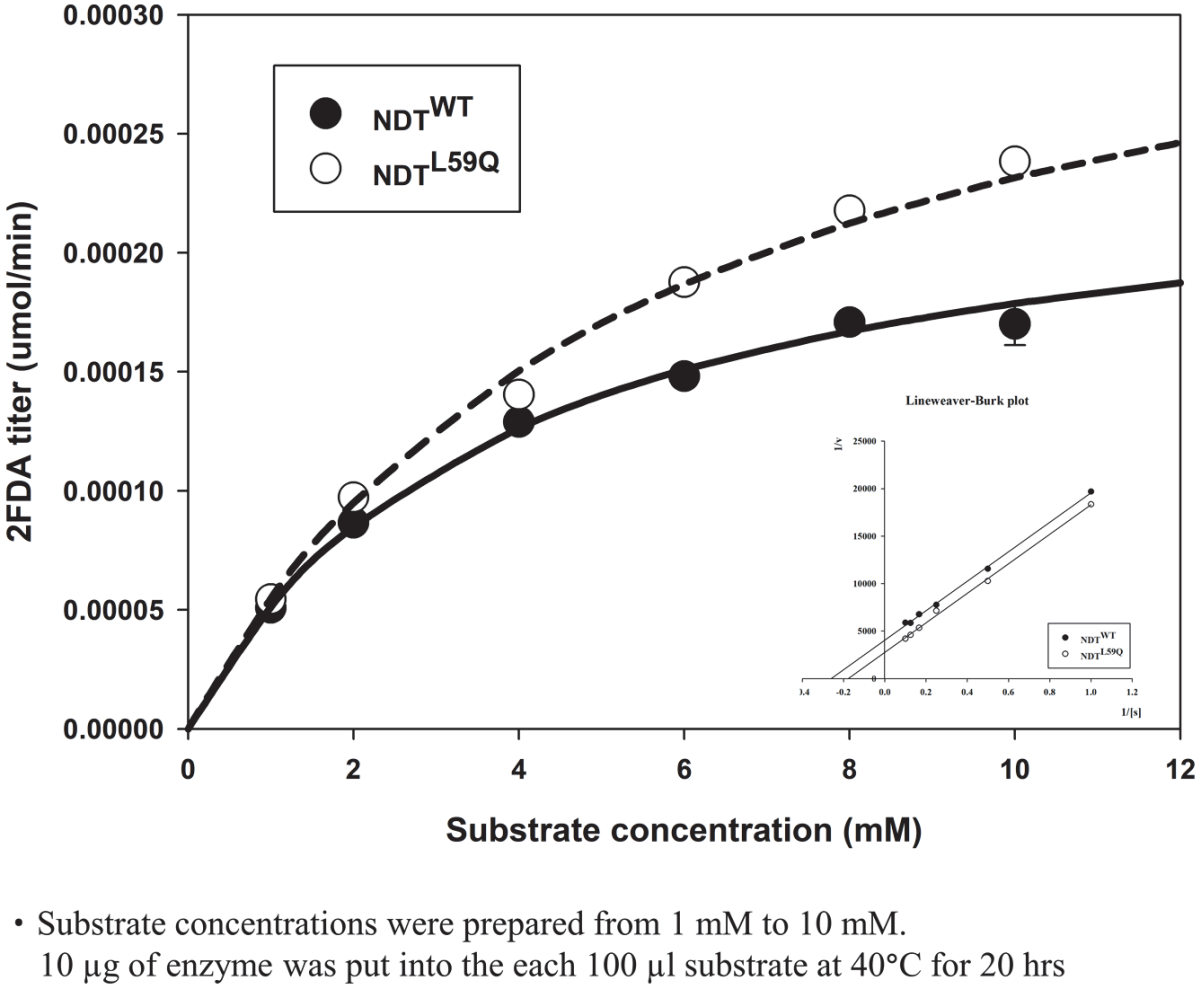
Kinetic parameters of NDT^WT^ and NDT^L59Q^. *V*_max_ and *K*_m_ values were determined by Lineweaver-Burk plot. The two graphs follow the Michaelis-Menten equation. Various substrate concentrations were selected for the reaction.

The kinetic values of NDT^WT^ and NDT^L59Q^ were obtained by conducting the experiment at different substrate concentrations (Fig. 5). The substrate was 2FDU as a sugar donor and adenine as a base donor. The Lineweaver–Burk plot was completed by taking inverse numbers of the substrate concentration and the amount of the product, respectively. Each value is listed in Table 2. In the case of the V_max_ value, purified NDT^L59Q^ was approximately 1.46 times higher than that of NDT^WT^, and the *K*_m_ value representing the affinity with the substrate was 3.847 mM for NDT^WT^, which was lower than that of 5.632 mM. NDT^WT^ was slightly higher in *K*_cat_/*K*_m_, a unit representing the efficiency of the enzymes. The kinetic values and characterization suggest that although the enzyme efficiency (*K*_cat_/*K*_m_) is similar, the specific activity of NDT^L59Q^ toward 2FDU was higher than NDT^WT^. With a wide range of temperatures, the stability of NDT^L59Q^ is believed to be the basis for this explanation. Moreover, the 2FDU substrate reacts for a relatively long time (18-20 h) compared to the other substrates, which is advantageous for NDT^L59Q^ in the industrial production of 2′-fluoro-2′-deoxynucleoside. Further NDT improvement strategies including bigger library screening as well as some site-specific mutagenesis are currently in progress. In summary, a rapid search for engineered enzymes that can synthesize nucleoside analogs can be done by generating random NDT mutant library via error-prone PCR, followed by confirmation of the products through one-pot reaction in the whole-cell conversion stage using *E. coli*-based crude extract. The strategy described here is expected to be applicable to screening other industrially valuable enzymes involved in nucleotide analogue bioconversion.

## Supplemental Materials

Supplementary data for this paper are available on-line only at http://jmb.or.kr.

## Figures and Tables

**Table 1:** 2FDU-to-2FDA conversion titers with several colonies obtained from error-prone PCR mutagenesis.

Colony	Nucleotide change	Amino acid change	Titer
A166	C33G	-	1.48
A292	A110G, A226G	176V	1.66
B131	A78G, G79A, A404G	D26E, A27T, K135R	1.29
B168	-	-	1.31
C22	C49A, A69G	E17K	2.12
C71	T176A	L59Q	2.39

**Table 2:** Kinetics values of NDT^WT^ and NDT^L59Q^.

Kinetics value	Purified protein	

	NDT^WT^	NDT^L59Q^
*V*_max_ (mM • hr^-1^)	4.95 •10^-3^ ± 0.268 •10^-3^	7.24 •10^-3^ ± 0.276 •10^-3^
*K*_m_ (mM)	3.847 ± 0.3331	5.632 ± 0.3161
*k*_cat_ (hr^-1^)	0.990 ± 0.0536	1.45 ± 0.0553
*k*_cat_/*K*_m_ (hr^-1^• mM^-1^)	0.258 ± 0.00869	0.257 ± 0.00484

•Substrate concentrations were prepared from 1 mM to 10 mM.

10 μg of enzyme was put into the each 100 μl substrate at 40°C for 20 hrs
