# Supplementary material for: Isolation and Characterization of Engineered Nucleoside Deoxyribosyltransferase with Enhanced Activity Toward 2’-Fluoro-2’-Deoxynucleoside
Source: J Microbiol Biotechnol. 2022 Jun 15;32(8):1041–6. doi: 10.4014/jmb.2204.04041 (PMC9628941; doi:10.4014/jmb.2204.04041)
Supplement: Supplementary file 1 [file jmb-32-8-1041-supple.pdf]

J. Microbiol. Biotechnol.
https://doi.org/10.4014/jmb.2204.04041

Supplementary Figures and Table

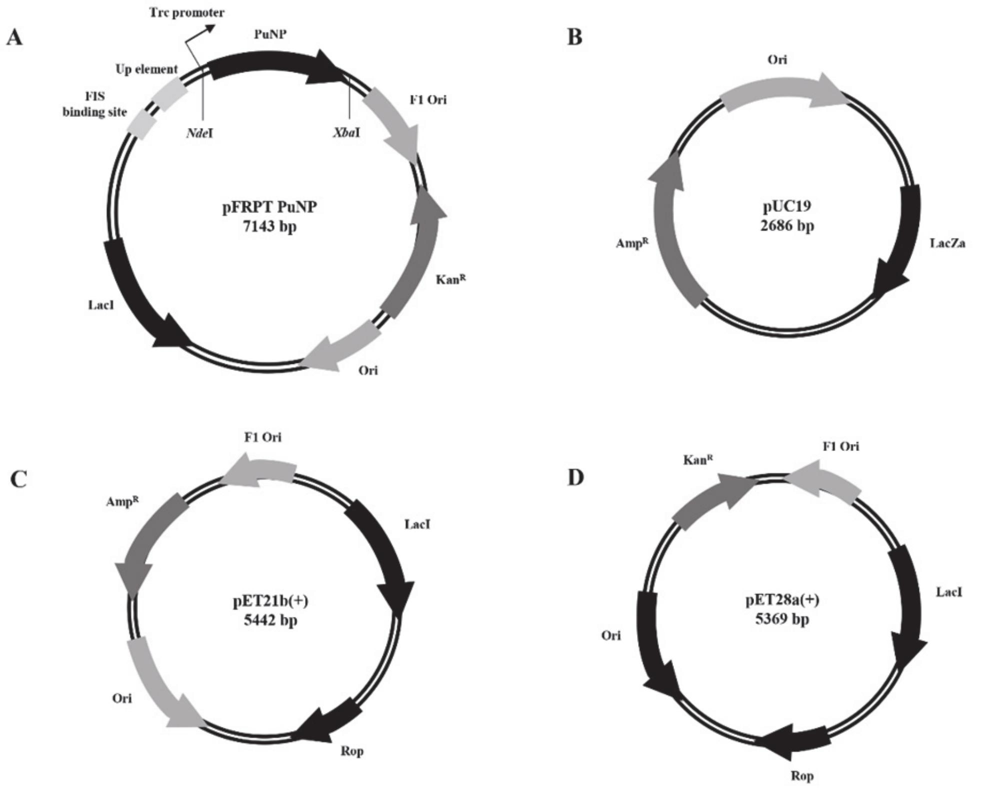

Figure S1. Plasmid list used in this study

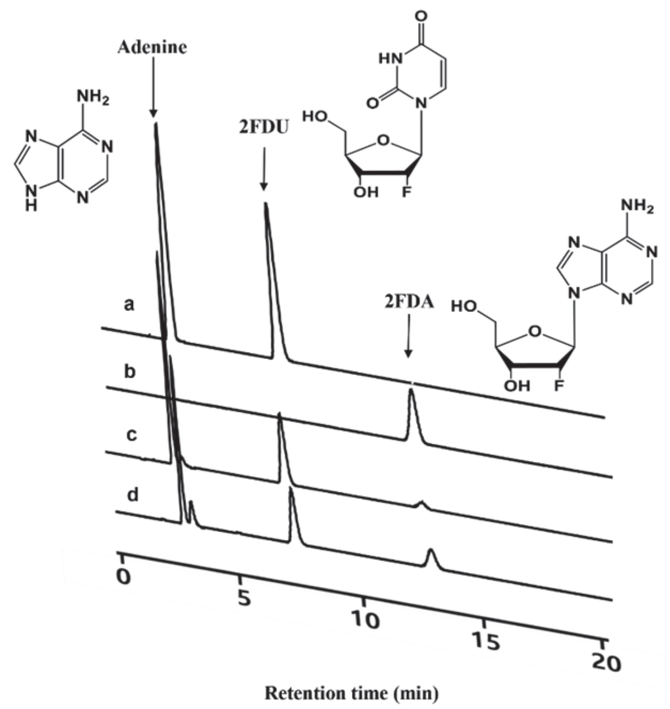

Figure S2. High performance liquid chromatography of 2FDU-to-2FDA conversion. HPLC chromatogram of (a) Adenine and 2FDU as standard substrates, (b) 2FDA as a standard product, (c) NDT^{WT} reacted with substrate, (d) NDT^{L59Q} reacted with substrate.

```
NDT^WT                      ------MPKKTIYFGAGWFTDRQNKAYKEAMEALKENPTIDLENSYVPLDNQYKGIRVDEHPEYLH-DKVWATATYNNDLNGIKTNDIMLGVYIPDEEDV
NDT^L59Q                    ------MPKKTIYFGAGWFTDRQNKAYKEAMEALKENPTIDLENSYVPLDNQYKGIRVDEHPEYQH-DKVWATATYNNDLNGIKTNDIMLGVYIPDEEDV
Lactobacillus_helveticus    -----MNKKKTLYFGAGWFNEKQNKAYKEAMAALKENPTVDLENSYVPLENQYKGIRIDEHPEYLH-NIEWASATYHNDLVGIKTSDVMLGVYLPEEEDV
Lactobacillus_reuteri       -----MNKKKTLYFGAGWFNEKQNKAYKEAMAALKENPTVDLENSYVPLENQYKGIRIDEHPEYLH-DIEWASATYHNDLVGIKTSDVMLGVYLPEEEDV
Lactobacillus_johnsonii     -------------MAGWFTETQNKAYKDAMSALNANPTIDLENSYVPLQNQYKDIRVDEHPEYLH-DKEWAQATYNGDLVGIKTSDVMLGVYVPKEEDV
Lactobacillus_fermetum      MKNTDPVANTKIYLATSFFNEEQRARIPQALAQLEANPTVGVVH--QPFDFQYKDARVDSDPAGVFGSLEWQIATYNNDLNAVGTSDVCVALYDMDQIDE
                                          :.:*.: *.    :*:  *: ***:.: :   *:: ***. *:*..*    . .  *  ***:.** .: *.*: :.:*  .: *

NDT^WT                      GLGMELGYALSQGKYVLLVIPD--EDYGKPINLMSWG---VSDNVIKMSQLKDFNFNKPRFDFYEGAVY-
NDT^L59Q                    GLGMELGYALSQGKYVLLVIPD--EDYGKPINLMSWG---VSDNVIKMSQLKDFNFNKPRFDFYEGAVY-
Lactobacillus_helveticus    GLGMELGYALSQGKYILLVIPD--EDYGKPINLMSWG---VCDNAIKISELKDFDFNKPRYNFYDGAVY-
Lactobacillus_reuteri       GLGMELGYALSQGKYILLVIPD--EDYGKPINLMSWG---VCDNAIKISELKDFDFNKPRYNFYDGAVY-
Lactobacillus_johnsonii     GLGMELGYAMSQGKYVLLVIPD--ELYGESINLMSWG---VADNVIKMSELATFDFNRPRYNFYDGAVY-
Lactobacillus_fermetum      GICMEIGMFVALHKPIVLLPFTKKDKSAYEANLMLARGVTTWLEPNDFSPLKDFNFNHPMAQPFPPFKVF
                            *: **:*  ::  * ::*:      :  .   ***      .  :  .:* *  *:**:*  : :
```

Figure S3. Multiple sequence alignment of the *ndt* from various strains Multiple sequence alignment by CLUSTALW. NDT^{WT}; *Lactobacillus leiichmannii* (uniprot code; Q9R5V5), NDT^{L59Q}; *L. helveticus ndt* (uniprot code; Q8RLY5), *L. reuteri ndt* (uniprot code; A5VK58), *L. johnsonii ndt* (uniprot code; Q74LQ9), *L. fermentum ndt* (uniprot code; Q6YNI5). Point of sequence change marked as red.

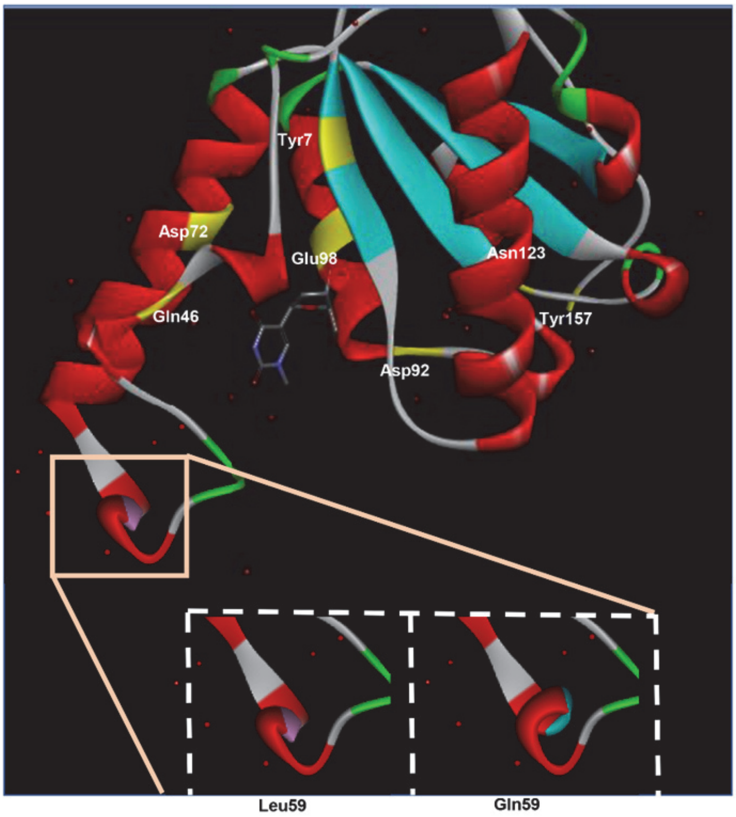

Figure S4. 3D structure of 1F8Y from *Lactobacillus leiichmannii* (*ndt*); PDB code 1F8Y. This figure shows the Interaction with deoxythymidine. The active site was highlighted in yellow.

Table S1. Primer list used in this study

Primer name	Sequence 5' -> 3'
NDTf	GATCATATGCCTAAGAAAACGATC
NDTr	CGTTCTAGATCTGCCCTTAATAAA
pFPRTf	GAGCGGATAACAATTCCCACC
pFRPTr	TCTCATCCGCCAAAACAGAAG
pUC19_NDTf	GCAGGTCGACTCTAGATGCCTAAGAAAACGATCTACTTCG
pUC19_NDTr	TGAGAGTGCACCATATTAATAAACGGCACCCTCGTAG
pET28a_NDTf	GGTGGTGGTGCTCGAGTTAATAAACGGCACCCTCGTAGAAGT
pET28a_NDTr	CGCGCGGCAGCCATATGCCTAAGAAAACGATCTACTTCGG
pET21b_NDTf	GGTGGTGGTGCTCGAGATAAACGGCACCCTCGTAGAAG
pET21b_NDTr	AAGGAGATATACATATGCCTAAGAAAACGATCTACTTCGG
